# Empirical demonstration of environmental sensing in catalytic RNA: evolution of interpretive behavior at the origins of life

**DOI:** 10.1186/s12862-014-0248-2

**Published:** 2014-12-04

**Authors:** Niles Lehman, Tess Bernhard, Brian C Larson, Andrew JN Robinson, Christopher CB Southgate

**Affiliations:** Department of Chemistry, Portland State University, Portland, OR USA; Department of Ecology and Evolutionary Biology, Princeton University, Princeton, NJ USA; College of Humanities, University of Exeter, Exeter, UK

**Keywords:** Interpretation, Signs, RNA, Ribozyme, Origins of life, Divalent cations

## Abstract

**Background:**

The origins of life on the Earth required chemical entities to interact with their environments in ways that could respond to natural selection. The concept of interpretation, where biotic entities use signs in their environment as proxy for the existence of other items of selective value in their environment, has been proposed on theoretical grounds to be relevant to the origins and early evolution of life. However this concept has not been demonstrated empirically.

**Results:**

Here, we present data that certain catalytic RNA sequences have properties that would enable interpretation of divalent cation levels in their environment. By assaying the responsiveness of two variants of the *Tetrahymena* ribozyme to the Ca^2+^ ion as a sign for the more catalytically useful Mg^2+^ ion, we show an empirical proof-of-principle that interpretation can be an evolvable trait in RNA, often suggested as a model system for early life. In particular we demonstrate that *in vitro*, the wild-type version of the *Tetrahymena* ribozyme is not interpretive, in that it cannot use Ca^2+^ as a sign for Mg^2+^. Yet a variant of this sequence containing five mutations that alter its ability to utilize the Ca^2+^ ion engenders a strong interpretive characteristic in this RNA.

**Conclusions:**

We have shown that RNA molecules in a test tube can meet the minimum criteria for the evolution of interpretive behaviour in regards to their responses to divalent metal ion concentrations in their environment. Interpretation in RNA molecules provides a property entirely dependent on natural physico-chemical interactions, but capable of shaping the evolutionary trajectory of macromolecules, especially in the earliest stages of life’s history.

## Background

The origins of life on the Earth can be described as a transition from an environment containing chemicals dominated by mass-action kinetics to one containing informational macromolecules dominated by selective forces and evolution [1,2]. The latter phenomena involve complex molecular ‘behaviour’ whereby populations of molecules compete for reproductive success. The interaction of organisms with an ever-changing environment has been a key evolutionary factor since the earliest entities emerged that were subject to natural selection [3]. It is thus of great importance to the understanding of the origin of life to understand the nascent stages of this interaction.

For a genotype (not necessarily originally a complete genome) to interact with its environment, it must first sense, and then respond to, environmental states. Primordial genotypes would not have been adept at these actions, as they would have not had the benefit of extensive evolutionary tuning. We contend that the most characteristic form of this sensing in living organisms (as opposed to inert material responding mechanically) is an interpretive process involving a *response* to a *sign*. At the molecular level, signs are indicators of a more general environmental condition; in particular here we will focus on metal ions as indicative of the presence or absence of other ions in the environment as a consequence of causally related solubilities in an aqueous milieu.

The idea that interpretation, representation, or signification is a fundamental characteristic of living things is the basis of the field of theoretical biology known as biosemiotics (semiotics being the philosophical study of signs) [4]. Biosemiotics is a controversial area of study because it has tended to lack empirically testable propositions. Our focus here is on providing just such opportunities for testing. Originally genotypes would be poor at correctly interpreting the state of the environment, but this trait would be subject to natural selection. To help explore how this evolution could happen, what was first needed was a conceptually robust definition of interpretation that cannot be reduced to a fully adequate re-description in terms only of mechanistic causes and effects. Though the interactions are entirely mechanistic when considered at the molecular level of description, a fully adequate explanation of why such properties would be selected and maintained by selection requires, in addition, the semiotic level of description. Two of the present authors have proposed such a definition [5].

In an interpretation, the trigger for the change of state (the ‘sign’) is related to, but not obligatorily implied by, some aspect of the environment (the ‘object’) that would make such a change in state beneficial. For example, an amoeba will crawl up a chemical gradient of attractant molecules towards the latter’s source. The chemotactic response is adaptive because it increases the chance of the amoeba obtaining nutrition (by ingesting the bacterium that is the source of the attractant). The response is interpretive because it is not the chemical gradient that is of benefit to the amoeba, but the bacterium of which the attractant molecules are a sign. The amoeba’s target (the bacterium) is physically distinct from the sign (the chemical attractant). It is this separation between sign and object that distinguishes an interpretation from a direct adaptive response; *i.e*., interpretations are *fallible*. The amoeba may swim up a chemical gradient that has not, in a particular case, been produced by a potential source of nutrition. In general, interpretive behaviour may be adaptive in spite of its fallibility because on balance the benefits derived from obtaining information about the object without having to respond directly to the object outweigh the costs of misinterpretations. The amoeba benefits from responding the attractant gradient because it can thereby detect the presence of a bacterium at a distance.

**Figure 1:**
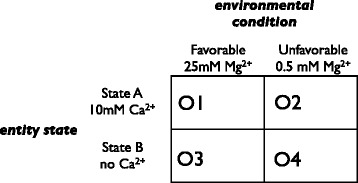
**Example payoff matrix for interpretive behaviour.** Payoff values **O1**–**O4** are evaluated for each pair-wise combination of environmental conditions and genotype traits as discussed in the text. The ion concentrations refer to those used in the assays of the *Tetrahymena* ribozyme, as described in Figures 2, 3 and 4.

The simplest conceivable scenario in which an adaptive interpretive response could occur would involve a two-state entity in a two-state environment [5]. Suppose that the environment can be ‘favourable’ (F) or ‘unfavourable’ (U) and the entity has two possible states, A and B. Suppose further that in environment F it is advantageous for the entity to be in state A, and in environment U it is advantageous for the entity to be in state B. The overall ‘fitness’ of the entity in this varying environment may be expressed, by analogy with game theory, in terms of a 2×2 payoff matrix (Figure 1). There are four possible *outcomes* in the matrix: **O1** (environment F, entity state A), **O2** (environment U, entity state A), **O3** (environment F, entity state B), and **O4** (environment U, entity state B). The total ‘payoff’ (overall fitness) for the entity is the sum of **O1** to **O4,** weighted according to the relative probabilities of each of these outcomes. In the non-interpretive configuration of the entity, its state (A or B) varies independently of the state of the environment. An interpretive variant of the entity might be capable of, say, responding to some sign that indicates (fallibly) that the state of the environment is F, the response being a change from state B to state A. This variant may have a selective advantage over the wildtype because it will increase the time that it spends in the advantageous combination of environment F and state A. Such interpretive responsiveness will only be adaptive, however, if it is not outweighed by the costs of a misinterpretation. This cost will be a function of the degree of disadvantage entailed by the entity being in state A in environment U, and the probability of being so placed in such a relation to the environment by the fallible (*i.e*., less than perfect) correlation between the presence of the sign and environmental state F.

For responsiveness to the sign to have the potential to count as an interpretation it is thus necessary that there be both a potential advantage to the response in the appropriate environment, and a potential disadvantage if the response occurs in the inappropriate environment (a misinterpretation). This may be expressed in terms of three inequalities that together provide the *minimum* criteria for interpretive behaviour in a 2-environment, 2-states-of-an-entity system. The relevant inequalities are: **O1** > **O2** (in state A the entity is better off in the favourable rather that the unfavourable environment); **O1** > **O3** (in the favourable environment the entity is better off in state A than state B); **O4** > **O2** (in the unfavourable environment the entity is better off in state B than state A, hence the *cost* of a misinterpretation) [6]. In addition, by definition, the highest of the four possible outcomes must correspond to the nominally favourable environment, otherwise that environment cannot be said to be the favourable one.

With this theoretical framework in hand, it then becomes imperative to demonstrate these phenomena in an empirical system. An obvious choice to manifest interpretation experimentally is ribonucleic acid (RNA), because RNA molecules simultaneously possess evolvability and catalytic function, and the RNA World concept is currently generally accepted as a plausible stage of early life on the Earth [1,7]. Specifically, we wished to test the hypothesis that a single biomolecule not part of a more complex cellular system may exhibit properties consistent with the minimum criteria for interpretive responsiveness. In this paper we report investigations of the properties of the group I self-splicing ribozyme from the ciliated protozoan *Tetrahymena*. As sign and object we use the availability of the divalent cations (M^2+^) which are not only prebiotically relevant but known to affect the catalytic behavior of ribozymes [8-10], and the response was a change in the catalytic rate of the ribozyme. We show that the cation dependency of the catalytic properties of a variant of this ribozyme could, under appropriate conditions in a hypothetical two-state environment, provide the basis on which the inequalities required as minimal conditions for interpretive responsiveness could be satisfied. We also demonstrate that such interpretive responsiveness can be an evolvable property in variants of this ribozyme, particularly in an origin-of-life context.

## Results

### Pick-up-the-tail assays

**Figure 2:**
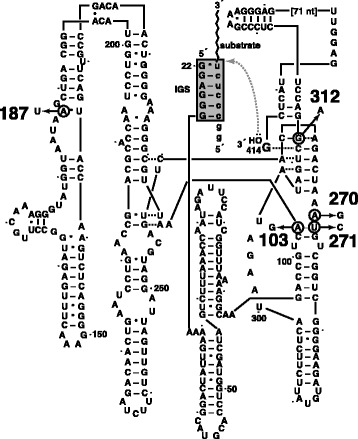
**The** ***Tetrahymena*** **ribozymes, with secondary structures modified from [** 10 **].** The L–21 (‘length minus 21’ nucleotides from the 5′ end of the *in vivo* intron) sequence is shown for the wild-type ribozyme, with the five mutations in the CaCl_2_-competent variant (PV) [12] indicated by the circles: A103G, A187U, A270G, U271C, and G312A. Numbering scheme follows the original [8,11,12], with dots provided every ten nucleotides. The interaction between the internal guide sequence (IGS) and six nucleotides of the substrate is shown by the grey box; in the ‘pick-up-the-tail’ assay the hydroxyl of the 3′ nucleotide (G414, shown) attacks at the splice site in the substrate (lower-case letters) and transfers the 3′ portion of the substrate (the last 17 nucleotides of S-1t in this case = AAAUAAAUAAAUAAAUA) to the 3′ end of the ribozyme, thereby lengthening it making it detectable by gel electrophoretic analysis. In this drawing, 71 nucleotide near the 3′ end of the ribozyme were omitted for clarity. Bars between nucleotide pairs denote canonical Watson-Crick pairs, while dots denote non-canonical base-paring interactions. The active site of this ribozyme is the environment around the G414 and the stack of base-triples that is above and below it [13].

**Figure 3:**
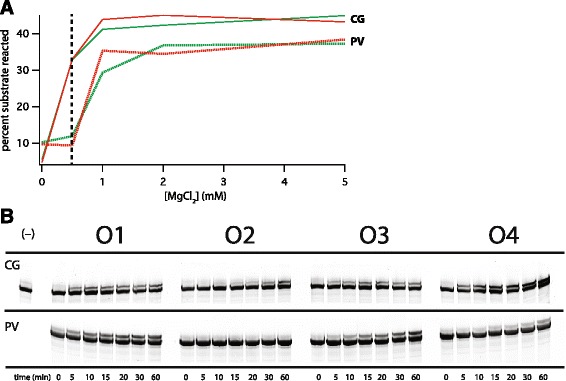
**Assays of the ribozymes in various divalent ion concentrations. (A)** Preliminary pick-up-the-tail assays were performed with 2.5-fold substrate excess as described in the text for activity after 60 minutes at 37°C. (Note: the original selection of the PV ribozyme [12] was performed at 3.25 h, at which time the PV activity surpasses that of the CG activity). The fraction of the initial amount of ribozyme RNA that reacted under conditions of 2.5-fold excess substrate was measured for each variant in the absence (green lines) or presence (red lines) of 10 mM CaCl_2_ under concentrations of MgCl_2_ ranging from 0.5–25 mM. Above 5 mM no increase in activity was observed. In the absence of a CaCl_2_ a slight inhibition of activity with increasing MgCl_2_ was observed above 0.1 mM, as noted before [8]. From these data, the conditions for formal analysis of interpretive behaviour were chosen near the endpoints of these curves: at, and well above, the point of maximum separation (0.5 mM MgCl_2_, indicated with the dashed black line). These conditions were 25 mM MgCl_2_ + 10 mM CaCl_2_ (**O1**), 0.5 mM MgCl_2_ + 10 mM CaCl_2_ (**O2**), 25 mM MgCl_2_ (**O3**), and 0.5 mM MgCl_2_ (**O4**). **(B)** Example gels of ribozyme reacted for 0–60 min with 2.5-fold excess S-1t substrate at 37°C (lower band: unreacted ribozyme; upper band: ribozyme reacted with S-1t substrate). Summarized data from all assays is shown in Table 1.

We chose two variants of the L–21 *Tetrahymena* ribozyme for study (Figure 2). The contemporary genotype (here, CG), an example of a present-day ‘wildtype’, is the L–21 derivative of the naturally occurring sequence obtained from *T. thermophila* and used in one of the first published examples of a catalytic RNA [11]. This ribozyme requires divalent cations for self-splicing or trans-splicing activity *in vitro*, and can utilize Mg^2+^ and/or Mn^2+^ but no other [8]. The ‘primitive variant’ (here, PV) is a five-error mutant of the wild-type ribozyme that was discovered through *in vitro* selection for activity in 10 mM CaCl_2_ (clone 61 in ref. [12]). This variant has a catalytic efficiency in pure CaCl_2_ that is five orders of magnitude less than that of the wild-type ribozyme (*i.e.*, the CG) in MgCl_2_, but its slight activity in Ca^2+^ is nonetheless notable because the ionic radius of Ca^2+^ is significantly larger than that of Mg^2+^, and the five mutations are required to open up the active site enough to confer detectable catalytic activity [10]. We tracked the ability of both the CG and PV versions of this ribozyme to catalyze phospho-ester transfer in four cation-specific buffer conditions. First we measured activity as a function of [MgCl_2_] and noted that there was a point of maximum catalytic separation between the two variants at 0.5 mM MgCl_2_ (Figure 3). From this we chose ‘high’ and ‘low’ concentrations of Mg to use in subsequent assays; these values were 25 mM and 0.5 mM respectively. These values have been shown before as being above and below, respectively, the midpoint of the *Tetrahymena* activity assay [8]. We chose 10 mM CaCl_2_ as the concentration of this alternative divalent (here, the sign) because this quantity is known to enhance folding of the wild-type *Tetrahymena* ribozyme in the presence or absence of MgCl_2_ [8] and is also the concentration at which the PV ribozyme was selected for activity in the absence of MgCl_2_ [12]. These ionic concentrations provide the clearest distinction between the properties of the two ribozymes and thus should optimize our chances of detecting interpretive behavior as an empirical proof-of-principle.

As seen previously, reaction progress as a function of time showed a rapid increase in product initially, with a plateau after approximately one hour. We measured the initial slope of the rapid reaction as a rough estimate of the initial velocity of the reaction to first approach the question of how well the ribozymes were performing under the various salt conditions. Given the extents of reactions of the two ribozymes under the ionic conditions chosen, we obtained the best estimates of initial velocities at 5 minutes for both ribozyme variants (see next section).

Using these initial slopes, the PV ribozyme satisfied all three inequalities needed for interpretive behaviour (**O1** > **O2**, **O1** > **O3**, and **O4** > **O2)** at all time points (*e.g*., Figure 3b). That is, in the favourable environment the PV ribozyme is better off in the presence than the absence of the ‘sign’ (Ca^2+^), and, notably, in the unfavourable environment the ribozyme is better off in the absence of the sign than in its presence, suggesting a potential evolutionary cost of misinterpretation (Table 1). We performed three replicates of each set of reactions and with the standard errors of the runs we used the Kruskal-Wallace rank-order statistic to determine that the ordering of the inequalities was marginally statistically significant for the PV at the *P* <0.1 level.

**Table 1:** **Percent reactivity values of the CG and PV ribozymes using the initial velocities of the pick-up-the-tail assays**

**Variant**	**O1**	**O2**	**O3**	**O4**	**O1 > ** **O2**	**O1** ** > O3**	**O4 > ** **O2**	**O1 > O4**
CG	18.8	9.24	14.2	31.1	yes	yes	yes	no
PV	14.7	1.46	2.42	2.37	yes	yes	yes	yes

For both ribozymes, the initial velocities were taken using data at 5 minutes. All data are the average of three replicates. Rank-ordering of the conditions are as follows. CG: **O4** > **O1** > **O3** > **O2** (not interpretive; *P* = 0.019 by Kruskal-Wallace test); PV: **O1** > **O4** > **O3** > **O2** (interpretive; *P* = 0.075 by Kruskal-Wallace test; note that this order differs from those given from the numbers in the table for the PV because they are averages from three trials).

The CG ribozyme also satisfied the three inequalities above. However, the highest performance of the CG ribozyme was in conditions of low [Mg^2+^] and the absence of the sign (Ca^2+^), *i.e*., outcome **O4**. By definition, for the CG ribozyme the favourable condition is therefore low rather than high [Mg^2+^], in which case the columns of the 2×2 matrix need to be reversed in order to assess whether the relevant inequalities are satisfied. When this is done the CG ribozyme does not meet the necessary criteria for interpretive behavior. This result does not depend on our choice of time points; the same trends are observed at any time up to 15 minutes (data not shown).

For the PV ribozyme, the **O3** and **O4** are relatively equal values. For this ribozyme, the ordinal ranking can be described as **O1** > **O3** ~ **O4** > **O2**. This is because the O3 *vs*. O4 ranking depends on which sets of data replicates are used, but the order between these two cells does not affect analysis of interpretive behavior. Thus for this variant, the presence of Ca^2+^ more dramatically alters the differentiation between the favorable and unfavorable states, allowing the ribozyme to better distinguish the disparity in Mg^2+^ content and hence be interpretive of its environment.

### Kinetic analyses with radiolabeled substrate

It was possible to estimate *k*_cat_ values from the assays performed with ^32^P-labeled substrate under conditions of enzyme excess for all scenarios except for the PV in **O2** and **O4** where the extents of reaction were not significant even after one hour. These values are summarized in Table 2. Here it can be observed that there is general agreement with the satisfying of the inequalities that constitute minimum criteria for the possibility of interpretive behaviour. However, because two of the PV values were not determinable, and because the enzyme excess conditions do not emulate those expected to drive the evolution of this trait, we did not rely on these data for our analysis of interpretation.

**Table 2:** **Rough estimates of** ***k***_**cat**_ **values under conditions of enzyme excess**

**Variant**	**O1**	**O2**	**O3**	**O4**	**O1** ** > O2**	**O1** ** > O3**	**O4** ** > O2**	**O1 > O4**
CG	7.0 × 10^−3^	2.3 × 10^−7^	5.5 × 10^−3^	5.5 × 10^−5^	yes	yes	yes	yes
PV	6.9 × 10^−3^	< 10^−8^	1.0 × 10^−3^	< 10^−8^	yes	yes	N.D.	yes

All values given in units of min^−1^. For the PV, yields under the **O2** and **O4** conditions were too small to estimate rate constants. Rank-ordering of the conditions are as follows. CG: **O1** > **O3** > **O4** > **O2**; PV: **O1** > **O3** > **O4 & O2**. However, the conditions of enzyme excess are not representative of an evolutionary interpretive scenario, hence the data in Table 1 were used for this purpose. N.D. = not determined.

**Figure 4:**
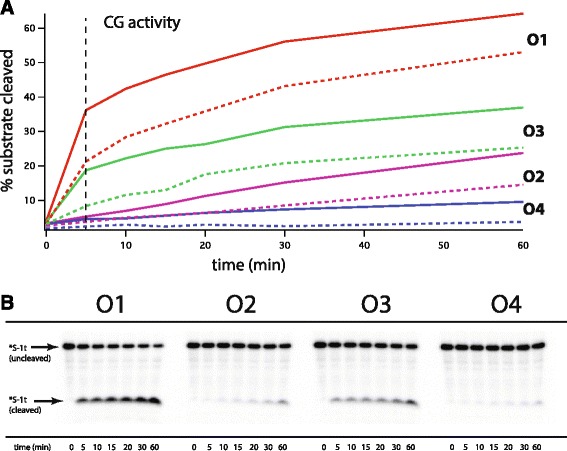
**Semi-formal kinetic analysis of ribozyme activities under the four environmental conditions O1–O4 using 5′-** ^**32**^ **P-radiolabeled substrate-cleavage assays. (A)** graph of percent substrate reacted as a function of time for the CG enzyme at two different concentrations: 25 nM (solid lines) and 10 nm (dotted lines). Horizontal dashed line indicates 5 min time point, where pick-up-the-tail data for interpretive analysis (Figure 3) was selected. **(B)** Example radiogram of cleavage patterns in the case of the CG ribozyme at 25 mM and *S-1t at 1 nM.

These ^32^P-labelling kinetic data were used to establish that the 5-minute time point was the optimal estimate of the initial velocities for the CG and PV ribozymes. From the extents of *trans*-splicing reactions of the ribozymes under the condition of enzyme excess over all four salt concentration combinations, we concluded that the CG consistently reacts approximately five times faster than the PV, and that the initial velocity values (where the extents of reaction are well differentiated yet below 50%) are best measured at 5 minutes, averaging over all four conditions (Figure 4). Moreover, the level of substrate cleavage under these assays was the highest for both ribozymes in the **O1** condition, which was not the case for the CG ribozyme under the pick-up-the-tail assay that tracked the behavior of the ribozyme itself. This result influenced our understanding of why the PV ribozyme is interpretive but the CG is not (see Discussion).

### Data analysis

An analysis of the total parameter space, and of the fitnesses of the PV ribozyme under study within that space, provides an opportunity to quantify interpretive behaviour of the ribozyme. As outlined in the Methods using equations (1), (2), and (3), the interpretive behaviour of the ribozyme can be quantified using an analysis of the total parameter space, defined by assigning values to three variables: the probability of high Mg^2+^ concentration (favourable environment), the probability of the presence of Ca^2+^ (presence of the sign) and the correlation, *r*, between the favourable environment and the presence of the sign. The proportion of total activity (fitness) of the ribozyme attributable to the property of interpretation is expressed by the interpretive index, *I*. If the total fitness of the ribozyme at a given point in parameter space is *W*_*t*_, and the fitness that would obtain if there were no correlation (predictive value) between the presence of the sign and the favourable environment is *W*_*c*_, then *I* = (*W*_*t*_ – *W*_*c*_)/*W*_*t*_ .

**Figure 5:**
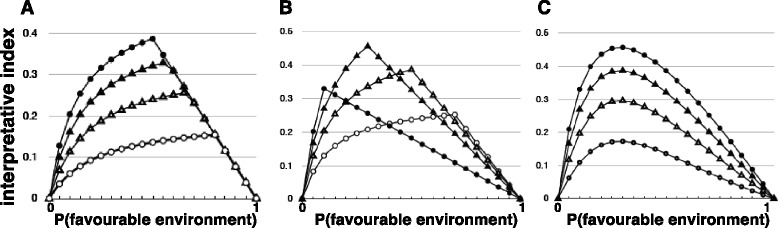
**Analyses of the interaction between interpretation and fitness for the PV ribozyme.** Data are for reaction times of 5 minutes. **(A)** The parameter-space in which the probability of the presence of the sign (10 mM Ca^2+^) is 0.5 and the probability of the presence of 25 mM Mg^2+^ (as opposed to 0.5 mM Mg^2+^) varies from 0 to 1 (*x*-axis). The proportion of total product attributable to the interpretive component (*I*) of the ribozyme activity is shown on the *y*-axis. Four different scenarios from the total parameter space are illustrated, each with a different correlation coefficient (*r*) between the probability of the sign (10 mM Ca^2+^) and the probability of the favourable environment (25 mM Mg^2+^): *r* = 1 (closed circles), *r* = 0.75 (closed triangles), *r* = 0.5 (open triangles), *r* = 0.25 (open circles). **(B)** The proportion of total activity of the PV ribozyme attributable to interpretation (*y*-axis) at varying probabilities of high (25 mM) Mg^2+^ (*x*-axis) with varying probabilities of the presence of the sign (10 mM Ca^2+^) and maximal correlation between the sign and the favourable environment (*r* = 1). Probability of the presence of the sign = 0.1 (closed circles), 0.3 (closed triangles), 0.5 (open triangles), 0.7 (open circles). For each of these curves a further set would be generated by lower levels of correlation, corresponding to the nest of curves in panel A. **(C)** Maximum available interpretive benefit (*y*-axis) at varying probabilities (*x*-axis) of the favourable environment (25 mM Mg^2+^) at various correlations (*r*) between the sign and the favourable environment: *r* = 1 (closed circles), *r* = 0.75 (closed triangles), *r* = 0.5 (open triangles), *r* = 0.25 (open circles). Each calculated point corresponds to a peak on a member of the set of curves of which four are shown panel B.

Figure 5a shows that, for the PV ribozyme, there is a positive interpretive component of fitness provided that there is a correlation between the presence of the sign (Ca^2+^) and the occurrence of the favourable environment (high concentration of Mg^2+^). As expected, the interpretive component of fitness rises with the degree of correlation between these variables.

Figure 5b shows the effect of varying the probability of the sign (presence of 10 mM Ca^2+^) when the correlation between the sign and the presence of the favourable environment (25 mM Mg^2+^) is maximal. Note that for a given probability of the favourable environment the maximal interpretive benefit occurs when the probability of the favourable environment is equal to the probability of the sign. This is as expected because it is the condition under which false positive and false negative responses to the sign are minimized. At higher levels of probability of the favourable environment the interpretive benefit is limited by the occurrence of false negatives (absence of the sign in the presence of the favourable environment). At lower levels of probability of the favourable environment the interpretive benefit is limited by false positives (presence of the sign in the absence of the favourable environment). (Figure 5a shows that the maximization of interpretive benefit when the probabilities of the sign and favourable environment are equal holds at all degrees of correlation between them).

Of the three parameter spaces represented in Figure 5b the greatest interpretive benefit is available where the probability of the favourable environment is close to 0.3. Calculations (not shown) reveal that the maximum occurs at a probability of the favourable environment of 0.33. The relationship between interpretive benefit and the probability of the favourable environment is explored further in Figure 5c, which shows the maximum interpretive benefit available at different probabilities of the presence of a favourable environment (*i.e*., parameter spaces in which the probability of the sign is equal to the probability of the favourable environment). As expected, interpretive benefit increases with increasing correlation between the sign and the interpretive environment.

## Discussion

The results of the assays above provide an empirical demonstration of interpretation as manifested at the molecular level. In addition, because the specific inequality ratios vary from one RNA sequence to another, we can infer that interpretation is an evolvable trait. When using the term ‘interpretation’ about a molecular property we are not suggesting any form of consciousness or deliberate choice; at the same time, from these results we do claim that a property more familiar in the behaviour of organisms is both definable and demonstrable at the molecular level [3].

We have shown that the PV ribozyme meets the necessary criteria for interpretive behavior. The maximum interpretive benefit (interpretive index) occurs at locations in parameter space at which the probabilities of the sign (presence of 10 mM Ca^2+^) and the presence of the favourable environment (25 mM Mg^2+^) are equal. This reflects the conditions under which the effects of false positive and false negative responses to the sign are minimized. The maximum interpretive benefit for the PV ribozyme at reaction times of 5 minutes occurs at the location in parameter space at which the probability of the favourable environment is 0.33. When the correlation between sign and favourable environment is maximal, and the probabilities of the sign and favourable environment are equal, the interpretive component of total fitness at this location in parameter space is 46% (Figure 5C). Even when the correlation is relatively low, *r* = 0.25, the interpretive component of total fitness is 17%. Levels of interpretive benefit sufficient to be subject to selection are also seen in less optimal areas of parameter space, in which the probabilities of the sign and favourable environment are not equal (Figure 5B). Importantly, the characteristics of the relationship between interpretive benefit and the parameter space variables illustrated in Figure 5 are properties of the whole system; *i.e*., they are ‘emergent’ properties of the 2 × 2 outcome matrix within the constraints defined by the values assigned to the parameter space variables. Therefore, although at a molecular level of description the physico-chemical interactions of a ribozyme molecule are adequate to explain its properties, a fully adequate explanation of a proto-biotic ribozyme sequence and structure might require reference to selection for interpretive behaviour, such an explanation necessarily involving reference to the ribozyme’s past or present relation to a probabilistically varying environment. We emphasize, however, that the PV variant of the *Tetrahymena* ribozyme represents merely a proof-of-principle in this regard: we are not suggesting that this particular ribozyme had any proto-biotic role.

In the absence of an analysis in terms of interpretation, the values observed for the artificially selected PV version of the *Tetrahymena* ribozyme in the payoff matrices would be somewhat surprising, because they show that the ribozyme does better in 0.5 mM MgCl_2_ (**O4**) than in the same situation but with 10 mM CaCl_2_ present (**O2**). This stands in contrast to the comparison between **O1** and **O3**, where it is seen that the addition of CaCl_2_ to a 25 mM MgCl_2_ buffer is advantageous. From a molecular perspective this can be explained through the realization that high amounts of a poorer cofactor (Ca^2+^) solution can flood the catalytic site and out-compete the preferred cation [8]. Thus the results seen here cannot be explained by a simple cooperative binding effect of one metal ion on another. Similarly, for the CG ribozyme, its low activity in **O1** can be attributed to the tendency of the *Tetrahymena* ribozyme to become trapped in unfavorable folded conformations at high, non-physiological concentrations of Mg^2+^ values [8,14]. This can be detected in the pick-up-the-tail assays where the ribozyme is being tracked but is not seen in the kinetic analyses of ^32^P-labeled substrate cleavage where there is vast enzyme excess (Figure 4).

But importantly, from the perspective of evolutionary fitness, the concept of interpretation adds a level of understanding of why certain patterns of cation utilization might be under selection. The concept of interpretation is therefore a significant and novel way of viewing evolutionary options in molecular systems. By considering that the sign (Ca^2+^) and the object (Mg^2+^) typically co-occur in biotic environments and in fact the former is typically at a concentration that is an order of magnitude higher than the latter (Table 3), an appreciation of a interpretive entity’s ability to exploit a chemically less proficient resource – and of the evolutionary advantage this brings – allows us to understand seemingly maladapted traits (*e.g*., here, **O4** > **O2**). This would be true in other molecular (and organismal) systems as well. It is worth emphasizing that we are not simply re-expressing the behaviour of the *Tetrahymena* chemical system in new language, but rather that we are positing that interpretation is a general evolutionary strategy operative from the molecular level upwards.

**Table 3:** **Comparison of the physical and geological characteristics of the two divalent cations under study as putative signs in this paper**

**Chemical properties**					**Modern conditions**		**Early life conditions**		
**Cation**	**Hydration number**	**Ionic radius (Å)**	***pK***_**a**_	**Hardness (η** ^**c**^ **)**	**Oceans (mM)**	**Cells (mM)**	**Archaean ocean (%)**	**Hydrothermal vents (mM)**	**Affinity for montmorillonite clay (** ***k***_**a**_ **/** ***k***_**Cd**_ **)**
Mg^2+^	6	0.72	11.42	47.59	50	30	24	51	0.9
Ca^2+^	8	1.12	12.70	19.52	10	1	29	232	5.8

Data taken from references [15-18].

**Figure 6:**
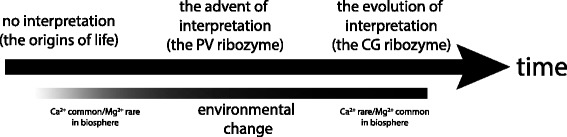
**Proposed evolutionary progression of interpretation in the** ***Tetrahymena*** **ribozyme system.** At or near the origins of life on the Earth 4 Ga, there were no catalytic RNAs with any interpretive ability. Selection for interpretation in a relatively calcium-rich but magnesium poor environment (Table 3) drove the advent of RNAs akin to the PV, which used Ca^2+^ as a sign for the preferred Mg^2+^ ion. As the abiotic and biotic environments evolved to sequester Mg^2+^ + ions in cells, the advantage to interpretation waned, and in contemporary cells relatively enriched in Mg^2+^ + RNAs such as the CG evolved under pressures to maximize their use of Mg^2+^.

The evolutionary scenario that we envisage from these results describes at least three distinct temporal stages (Figure 6). The first stage would be that of primordial, nearly random, and simple RNA sequences not optimized for replicative activity by natural selection. These RNAs would take advantage of the dominant divalent cation in their environment (Ca^2+^) to facilitate catalysis [19]. The binding would have been weak and many RNA sequences would have been able to conform to the binding of the relatively large and diffuse Ca^2+^ ion; but any anionic backbone shielding and/or catalytic prowess provided by this ion would have been potentially beneficial. Here the Ca^2+^ ion would have been the object itself, and not a sign.

The second stage would have been the advent of ribozymes with interpretive behavior, and the PV is seen as offering a proof-of-principle analogy for the actual RNAs that could have evolved during this time period. Here, the advantage of using Ca^2+^ as a sign in environment where the presence of Ca^2+^ and Mg^2+^ was correlated would imbue such sequences with a fitness advantage as they could accommodate both ions but make use of the chemically more powerful Mg^2+^ ion when available. More specifically, the structural complexity of the RNA had reached a point where the number and conformation of its cation biding sites allowed for a simultaneous distinction between, yet rudimentary simultaneous usage of, both Ca^2+^ and Mg^2+^. The ribozyme can use the former as a sign for the latter, and we expect that simulation models will later show that this feature is enhanced in environments where the appearance of these two ions is correlated in time and space. At this evolutionary stage interpretive molecular behaviour would be advantageous and would be under positive selection.

The third stage would have been the evolution of highly specific ribozyme sequences (and their corresponding 3° structures) able to form highly specific and tight active sites that could make maximal use of the preferred Mg^2+^ ion. These sequences (*e.g*., the CG variant that is extant in biology today) would have the benefit of long periods of evolutionary optimization in a defined cellular environment where ion transporters could use energy to pump ions against an environmental concentration gradient and create an optimal internal milieu, such as one that is enriched in Mg^2+^ compared to Ca^2+^. Here the evolutionary pressure for this particular kind of molecule would be for maximal and controllable catalytic activity, rather than for the ability to take advantage of signs of the state of the external environment, and thus interpretive behaviour would have been diminished or lost as it would no longer confer any selective advantage. Of course the use of signs (more often referred to as messages or signals in biological contexts) remains an important aspect of intracellular and inter-cellular communication. Our point is that in certain circumstances there may be a selective benefit to interpretive behaviour at one stage of evolution which is then superseded by a more efficient non-interpretive strategy based on high specificity binding. It is even conceivable that biotic systems ultimately retained the use of primordial signs such as Ca^2+^ for intercellular messaging purposes where the ion (sign) now has an arbitrary relation to object (intracellular or extracellular state) that it signifies.

It may be the case that other biopolymers such as proteins could have experienced evolutionary pressures based on interpretive abilities. Yet the potential central role of RNA in early life as a consequence of its dual genotypic and phenotypic functionalities makes it an ideal candidate for searching for interpretive behaviour. We anticipate that this characteristic could be evident in other RNA systems, particularly RNAs with known alternative folded structures, such as riboswitches, as discussed previously [20]. It may also be the case that other signs and objects could be utilized by RNA in an interpretive fashion. The divalent cations Mg^2+^ and Ca^2+^ are both alkali earth metals with similar, but not identical ionic radii (Table 3), and thus display many of the same chemical properties such as aqueous solubility and mineralization with subtle but often critical differences. This made them likewise good candidates for sign/object partners, and the well-studied metal-dependence [8,10,12] of the *Tetrahymena* ribozyme made it a logical system in which to investigate interpretive behaviour. Other ions such as Mn^2+^ [8] and Fe^2+^ [21,22] are also of interest in this regard, given the roles they can play in RNA-based catalysis.

In a system in which the definition of interpretation and the three inequalities discussed above are all satisfied, it is worth considering how such an interpreting entity might evolve further. Such further evolution might include: response to a different sign that is more highly correlated with the ‘object’ (the entity of which the sign is functioning as a sign); greater specificity of binding of the sign (reducing errors involving analogues of the sign); strategies for reducing the disadvantage of binding the sign when the object is not present; or, in some cases, enhanced capacity to recognize the object, such that recognition of the sign is no longer advantageous. (The latter scenario is the one that we envisage could explain the evolution of the current genotype, CG, of the *Tetrahymena* ribozyme from the primitive variant, PV.) We propose that it should be possible to observe each of these possibilities by performing targeted artificial evolution experiments on ribozymes in which interpretation has been demonstrated. From the last possibility it may be postulated that interpretations at the molecular level may be evolutionarily more primitive than high-specificity binding, and may have been more widespread than the latter in the early stages of the evolution of life.

## Conclusions

Using variants of the *Tetrahymena* ribozyme, we have shown that they can meet the minimum criteria for the evolution of interpretive behaviour in regards to their responses to divalent metal ion concentrations in their environment. In particular, one variant is interpretive, while another (the contemporary sequence) is not, suggesting that an evolutionary transition can take place and that interpretiveness can be under the influence of selection, either directly in a highly variable or unpredictable environment, or indirectly as in a laboratory selection for a specific phenotype. Interpretation must take place through a physical-chemical interaction between larger genotype molecules and smaller sign molecules; most likely binding constants at catalytic, general folding, or specific allosteric sites on genotypic polymers will prove to be the critical factors. Computer simulations of such events and/or additional examples of putative interpretive behavior in other RNAs will be quite valuable. Interpretation in RNA molecules provides a distinct type of intermolecular interaction event that can shape the evolutionary trajectory of macromolecules, especially in the earliest stages of life’s history.

## Methods

### RNA preparation

The variants of the 393-nucleotide *Tetrahymena* ribozyme were transcribed *in vitro* from DNA templates as described previously [10]. The two ribozymes were purified by electrophoresis through 8% polyacrylamide / 8 M urea gels and resuspended in 0.1 mM EDTA at 10 pmol/μl concentration prior to use. The *trans*-splicing substrate for *in vitro* assays, the 25-mer RNA ‘S-1t’ [23]: 5′-GGCCCUCU•AAAUAAAUAAAUAAAUA-3′, was purchased commercially from IDT (Coralville, IA). Upon reaction with the *Tetrahymena* ribozyme, this molecule is spliced at the dot (•) such that the 3′ end is transferred to the 3′ end of the ribozyme and the 5′ end is liberated.

### Ribozyme assays

The initial assays were performed in a ‘pick-up-the-tail’ format [12,24] in which the reaction was followed by tracking the length increase of the ribozyme following addition of the 3′ end of the substrate molecule. Successful reaction between the L–21 variants of the *Tetrahymena* ribozyme and the S-1t substrate results in a length change from 393 to 410 nt, which can be followed electrophoretically. For assays, 0.5 μM ribozyme was incubated with 1.25 μM substrate for 0–60 min in four different reaction buffers, all of which were buffered to pH 8.0 with 30 mM EPPS. Previously we tracked the reactivity in 0.5–50 mM MgCl_2_ after 195 min (Figure 4 in ref. [12]), and from this we opted to perform subsequent studies for shorter times (0–15 min; to address better the ribozymes’ initial velocities) in the following four reaction buffers (final concentrations): 25 mM MgCl_2_ + 10 mM CaCl_2_ (**O1**), 0.5 mM MgCl_2_ + 10 mM CaCl_2_ (**O2**), 25 mM MgCl_2_ (**O3**), and 0.5 mM MgCl_2_ (**O4**). These concentration values are similar to, but outside of the range of, those investigated earlier [8] for the CG ribozyme. (The existence of a Ca^2+^-competent mutant was not known when that study [8] was performed.) Reactions were stopped by the addition of enough EDTA to chelate stoichiometrically all divalent cations in solution, and the products were electrophoresed through 8% polyacrylamide / 8 M urea gels. The resulting bands were detected and quantified after staining with SybrGreen II on a Typhoon Trio^+^ imager and quantified using the ImageQuant software (GE Healthcare). The small but clear difference between the reactant (393 nt) and the product (410 nt) allowed the employment of SybrGreen staining with small error. All reactions were performed in triplicate.

### Kinetic studies

While the assays described in the previous section were entirely satisfactory (see Results) and performed under conditions of substrate excess, which would be pre-biotically relevant if one considers that nascent RNA genotypes would be far outnumbered by any substrate or “food” molecules, we performed additional analyses under conditions of ribozyme excess for the two ribozyme variants under differing environmental conditions following established protocols [10,12]. Here, the reaction was followed by tracking the length loss of the substrate following transposition of its 3′ end to the ribozyme and subsequent release of the 5′ portion, which was radiolabeled with γ-^32^P[ATP] by the use of the Optikinase enzyme (USB Biochemicals, Cleveland, OH). For assays, 10 or 25 nM ribozyme was incubated with 1.0 nM substrate for 0–60 min in the four different reaction buffers **O1**–**O4** described above. During this period, with this stoichiometry, less than 50% (and usually less than 20%) of the substrate has been converted to product such that single turnover conditions were maintained. Reactions were stopped by the addition of enough EDTA to chelate stoichiometrically all divalent cations in solution, and the products were electrophoresed through 16% polyacrylamide / 8 M urea gels and the resulting bands were detected by phosphorimaging on a Typhoon Trio^+^ imager and quantified using the ImageQuant software (GE Healthcare). The apparent rate constants *k*_*obs*_ were estimated by assuming that substrate binding was not rate limiting and that the reaction could be approximated with by first-order kinetics. While not strictly true for the *Tetrahymena* ribozyme under lower salt and higher temperature conditions (10 mM MgCl_2_ and 50°C) [14], this assumption is likely a good one averaged over all four reaction conditions studied here where the salt concentrations are typically higher and the temperature lower (37°C). The values of *k*_obs_ were obtained by fitting the fraction substrate cleaved (*f*) data to the equation *f* = *A*{1–exp(−*k*_obs_*t*)}, where *A* is the asymptotic maximum extent of reaction. The pseudo-first-order rate constants *k*_cat_ were obtained estimating the *y*-intercept of plots of *k*_obs_ vs. *k*_obs_/[E] for each ribozyme in each condition (**O1**–**O4**). Because the vast ribozyme excess did not emulate conditions expected for interpretive scenarios, these data were primarily used to establish the best time point to assay the ribozymes for interpretive behaviour using data from the previous section (see Results), and only secondarily to assess interpretive behaviour.

### Parameter-space analysis

An analysis of the total parameter space, and of the fitnesses of the PV ribozyme under study within that space, provides an opportunity to quantify interpretive behaviour of the ribozyme. The parameter space is defined by assigning values to three variables: the probability of high Mg^2+^ concentration (favourable environment), the probability of the presence of Ca^2+^ (presence of the sign) and the correlation between the favourable environment and the presence of the sign.

The proportion of total activity (fitness) of the ribozyme attributable to the benefit of interpretation is calculated as follows. The total activity (fitness) of a ribozyme (*W*_*t*_) at any location within this parameter space is given by the sum of the four experimentally measured outcomes in the 2 × 2 matrix (**O1**, **O2**, **O3**, and **O4**), weighted according to the probability of each outcome, p(**O1**), p(**O2**), p(**O3**), p(**O4**):

1 $$ {W}_t=\mathbf{O}\mathbf{1}\bullet \mathrm{p}\left(\mathbf{O}\mathbf{1}\right) + \mathbf{O}\mathbf{2}\bullet \mathrm{p}\left(\mathbf{O}\mathbf{2}\right) + \mathbf{O}\mathbf{3}\bullet \mathrm{p}\left(\mathbf{O}\mathbf{3}\right) + \mathbf{O}\mathbf{4}\bullet \mathrm{p}\left(\mathbf{O}\mathbf{4}\right) $$

The component of total fitness attributable to interpretation at any location in the parameter space (the interpretive index) can be quantified by correcting for the activity calculated for the ribozyme expected in the absence of any interpretive benefit. This ‘control’ fitness (*W*_*c*_) is given by the total fitness that would obtain if there were, in fact, no correlation between the presence of the sign and the favourable environment: *i.e*., if the probabilities of these were independent. If the probability of the sign is p(S) and the probability of the favourable environment is p(E), then:

2 $$ {W}_c=\mathbf{O}\mathbf{1}\bullet \mathrm{p}\left(\mathrm{S}\right)\mathrm{p}\left(\mathrm{E}\right) + \kern0.5em \mathbf{O}\mathbf{2}\bullet \mathrm{p}\left(\mathrm{S}\right)\left(1\hbox{--} \mathrm{p}\left(\mathrm{E}\right)\right)+\kern0.5em \mathbf{O}\mathbf{3}\bullet \left(1\hbox{--} \mathrm{p}\left(\mathrm{S}\right)\right)\mathrm{p}\left(\mathrm{E}\right) + \kern0.5em \mathbf{O}\mathbf{4}\bullet \left(1\hbox{--} \mathrm{p}\left(\mathrm{S}\right)\right)\left(1\hbox{--} \mathrm{p}\left(\mathrm{E}\right)\right) $$

When the correlation, *r*, between the presence of the sign and the presence of the favourable environment is zero, the probability of their co-occurrence, p(**O1**), is given by p(S)•p(E). In that case *W*_*t* =_*W*_*c*_ and the presence of the sign carries no predictive value with respect to the presence of the favourable environment_._ When the correlation is maximal, *r* = 1, the probability of the co-occurrence of sign and favourable environment, p(**O1**) is equal to the lower of p(S) and p(E). Between this minimum and maximum, the probability of co-occurrence of sign and favourable environment is linearly related to the correlation coefficient, *r*. For any value of p(**O1**) within parameter space, the values of p(**O2**), p(**O3**) and p(**O4**) are determined by the fact that p(S) = p(**O1**) + p(**O2**) and p(E) = p(**O1**) + p(**O3**).

The proportion of total fitness attributable to interpretive benefit (the interpretive index, *I*) is then given by:

3 $$ I = \left({W}_t\hbox{--} {W}_c\right)\ /{W}_t $$
